# Determining the Composite Structure of Au-Fe-Based Submicrometre Spherical Particles Fabricated by Pulsed-Laser Melting in Liquid

**DOI:** 10.3390/nano9020198

**Published:** 2019-02-03

**Authors:** Hokuto Fuse, Naoto Koshizaki, Yoshie Ishikawa, Zaneta Swiatkowska-Warkocka

**Affiliations:** 1 Graduate School of Engineering, Hokkaido University, Sapporo, Hokkaido 060-8628, Japan; ttx-ken@frontier.hokudai.ac.jp; 2 Nanomaterials Research Institute, National Institute of Advanced Industrial Science and Technology (AIST), Tsukuba, Ibaraki 305-8565, Japan; ishikawa.yoshie@aist.go.jp; 3 Institute of Nuclear Physics, Polish Academy of Sciences, PL-31342 Kraków, Poland; swiatkow@wp.pl

**Keywords:** laser melting in liquid, Au-Fe alloy, submicrometre spherical particles, phase separation, reaction control, core-shell particles, laser wavelength, zeta potential

## Abstract

Submicrometre spherical particles made of Au and Fe can be fabricated by pulsed-laser melting in liquid (PLML) using a mixture of Au and iron oxide nanoparticles as the raw particles dispersed in ethanol, although the detailed formation mechanism has not yet been clarified. Using a 355 nm pulsed laser to avoid extreme temperature difference between two different raw particles during laser irradiation and an Fe_2_O_3_ raw nanoparticle colloidal solution as an iron source to promote the aggregation of Au and Fe_2_O_3_ nanoparticles, we performed intensive characterization of the products and clarified the formation mechanism of Au-Fe composite submicrometre spherical particles. Because of the above two measures (Fe_2_O_3_ raw nanoparticle and 355 nm pulsed laser), the products—whether the particles are phase-separated or homogeneous alloys—basically follow the phase diagram. In Fe-rich range, the phase-separated Au-core/Fe-shell particles were formed, because quenching induces an earlier solidification of the Fe-rich component as a result of cooling from the surrounding ethanol. If the particle size is small, the quenching rate becomes very rapid and particles were less phase-separated. For high Au contents exceeding 70% in weight, crystalline Au-rich alloys were formed without phase separation. Thus, this aggregation control is required to selectively form homogeneous or phase-separated larger submicrometre-sized particles by PLML.

## 1. Introduction

Pulsed-laser melting in liquid (PLML) is a technique derived from pulsed-laser ablation in liquid (PLAL) for nanoparticle fabrication [1,2]. In PLML, raw nanoparticles dispersed in liquid are irradiated by a pulsed-laser with a moderate fluence of about 50–200 mJ pulse^−1^ cm^−2^ (lower fluence than PLAL), resulting in melting and fusion of irradiated particles and subsequently the formation of submicrometre-sized spherical particles via cooling [3,4,5,6]. PLML can produce submicrometre spherical particles of various materials, such as metals [7,8], oxides [5,6,9], semiconductors [10,11] and carbides [3,4]. Given the unique features of submicrometre spherical particles—including their dispersibility, stability, crystallinity and sphericity—applications utilizing optical [5,12], medical [13], mechanical [14,15] and magnetic [16] functionality have been examined.

From single-component raw particles, spherical particles are formed without compositional change simply by melting [6]. Reactive fabrication of submicrometre spherical particles with a different composition than the raw particles has been reported for B_4_C from B [3,4], Cu from CuO [8] and Fe and FeO from Fe_3_O_4_ [17] by a reaction with surrounding organic solvents. Other approaches for forming submicrometre spherical particles of alloys have been intensively investigated from two-component raw particle mixtures, especially for alloy systems of Au and transition metals like Fe, Co and Ni [18,19,20,21,22,23]. For example, an Au-Co alloy was formed by PLML, although the Au-Co combination was immiscible and could not form an alloy by conventional thermochemical processes [19]. This non-equilibrium process is due to the unique nature of the heating and quenching processes in PLML. In particular, the space-selective pulsed heating of PLML is completely different from conventional heating processes, such as furnace heating. In PLML, the temperature surpasses the melting point of the particles in several hundreds of nanoseconds or shorter, with heating and cooling rates of 10^11^ K s^−1^ and 10^10^ K s^−1^, respectively [24]. Since pulsed lasers with repetition rate of 10–100 Hz are generally used for PLML, these rapid heating and quenching cycles are repeated many times, with an interval of 10–100 ms for cooling process [25]. Liquid phase surrounding particles acts as a heat dissipation barrier after temporal vaporization and a cooling medium for quenching.

In particular, Au-Fe bimetallic particles have recently been attracting considerable interest because they can be multifunctional materials, combining the plasmonic properties of Au and the magnetic properties of Fe [26,27,28]. In addition, they may combine to have synergistic functions, such as oxygen evolution, enhanced plasmon absorption, carbon dioxide reduction and imaging and photo-thermal therapy [29,30,31,32]. Therefore, various fabrication techniques and formation mechanisms of Au-Fe nanoparticles prepared by PLAL have been intensively investigated [33,34,35,36,37] because the technique’s contamination-free nature is beneficial for biological and medical applications. 

However, extensive studies on submicrometre-sized Au-Fe particles have not been conducted due to the limited availability of suitable fabrication methods [38]. Our group previously tried to fabricate Au-Fe submicrometre spherical particles by PLML using the second harmonics of an neodymium-doped yttrium aluminium garnet (Nd:YAG) laser at a wavelength of 532 nm [18,22]. Raw particles dispersed in ethanol (C_2_H_5_OH) were chemically synthesized Fe_3_O_4_ and laser-ablated Au nanoparticles. During laser irradiation, Fe_3_O_4_ nanoparticles were reduced by ethanol to FeO or Fe, depending on the laser irradiation conditions. Reduced Fe particles were merged with Au nanoparticles to form submicrometre-sized alloy or composite particles. The heating, reducing, merging, alloying and spheroidizing processes occurred concurrently and submicrometre-sized spherical particles were formed. The magnetic properties of the products were also reported. 

Heating behaviour of single particle can be discussed via thermal diffusion length during pulsed laser heating [39]. For 7 ns (pulse width) laser irradiation, thermal diffusion length can be calculated to be 1340 nm for Au fairly larger than the particle size. Thus, even if skin layer of Au particle are selectively heated, the temperature is easily homogenized within Au particle in 7 ns by laser heating. Although the thermal diffusion length of iron oxide is rather difficult to be estimated due to the lack of reliable thermal diffusivity data, it can be roughly estimated to be 350 nm. Thus, the temperature within a single particle can be promptly homogenized during nanosecond laser irradiation. 

**Figure 1:**
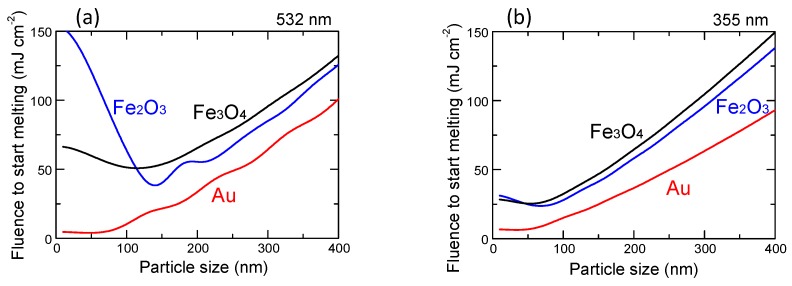
Particle size dependence of laser fluence required to melt a single particle of Au, Fe_3_O_4_ and Fe_2_O_3_ calculated based on Mie theory and adiabatic assumption. (**a**) 532 nm. (**b**) 355 nm.

However, in two-component raw particle systems, the different heating behaviours of each component particle caused by the difference in optical absorption efficiency affects the initial heating step and hence the morphology and inner structure of the products. Figure 1a shows the particle size dependence of the laser fluence required to melt a single particle of Au, Fe_3_O_4_ and Fe_2_O_3_ via irradiation with a 532 nm laser light, as calculated based on Mie theory under an adiabatic assumption [40,41]. Although Au has strong optical absorption at this wavelength due to surface plasmon resonance, Fe_3_O_4_ requires a higher fluence of more than 50 mJ for smaller particles (<100 nm). In contrast, at a wavelength of 355 nm (Figure 1b), the difference in fluence to melt a single particle of each component is less. Thus, the extent of inhomogeneous heating and resultant extreme temperature difference between the respective raw particles is reduced by changing the laser wavelength from 532 nm to 355 nm.

The immediacy of two constituent particles for a prompt reaction during laser irradiation is also an important factor for two-component raw particle systems. Fabricating large, submicrometre spherical particles from raw small nanoparticles during a short laser irradiation time requires such aggregation. In alloy particle formation processes, two different, individually fabricated raw particle solutions have to be mixed. Once dispersed, particles have to encounter one another and combine to form aggregates composed of two different particles. Otherwise, a reaction between the two components would not effectively occur in a short time. 

In our previous experiments, chemically produced Fe_3_O_4_ particles were used as an iron source and the zeta potential of the Fe_3_O_4_ raw particles fluctuated from −44 to −94 mV in a relatively large negative value. Au nanoparticles fabricated by PLAL are well known to have large negative zeta potentials without any surfactant [42]. Thus, after mixing, these two components are stably dispersed in liquid without a strong interaction between the two raw particles. If positively charged raw particles are available as an iron source, the aggregation proceeds after the raw particles mix because of an electrostatic interaction. Such a source is beneficial for the formation of submicrometre spherical particles based on well-reacted constituent particles.

Here, in contrast to our previous work, a 355 nm laser was adopted for more homogeneous heating of the raw particles and raw Fe_2_O_3_ particles with positive zeta potential were used for a stronger interaction with negatively charged raw Au nanoparticles. In investigating Au-Fe alloy submicrometre spherical particle fabrication, we especially focused on the detailed concentration-dependence of the products to understand what governs the reaction process in this technique. We also compared our experimental results with those obtained using PLAL for Au-Fe nanoparticle systems. In our discussion, we clarify the procedure to produce particles with thermodynamically stable phases or those with unexpected immiscible alloy phases.

## 2. Materials and Methods 

Raw Au particles were prepared by pulsed-laser ablation of Au plates as a target immersed in ethanol and irradiation with the fundamental wave (1064 nm) of the Nd:YAG laser for 60 min. The average Au particle diameter was estimated to be 10 nm from the relationship between laser fluence and particle size. Raw particles of Fe_2_O_3_ were purchased from Sigma-Aldrich Japan (Tokyo, Japan, product number: 544884, <50 nm average diameter). 

Raw nanoparticles of Au (140 ppm) and Fe_2_O_3_ (200 ppm) dispersed in ethanol were mixed with different weight ratios of particles in Au:Fe—10%:90%, 20%:80%, …, 90%:10% in every 10% step. Hereafter, we denote these mixtures as Au10, Au20, …, Au90, respectively. The third harmonic of the Nd:YAG laser (wavelength: 355 nm, repetition rate: 10 Hz, pulse width: 7 ns) was used to irradiate the mixed solutions (5 mL) with a fluence of 150 mJ pulse^−1^ cm^−2^ for 60 min so that the compositional effect on the resulting products could be studied. The total number of irradiated pulses to the colloidal solution is 3.6 × 10^4^ in this experiment, though the actual irradiated pulses to each particle is around hundredth or thousandth of irradiated pulses due to shadowing effect of laser-absorbing particles and stirring conditions. This laser fluence is sufficiently large to melt Au and Fe_2_O_3_ raw particles, as well as the produced submicrometre particles (Figure 1b). In addition, to analyse the effect of irradiation time, we irradiated the mixed nanoparticle solution for Au50 for 10, 30, 60 and 120 min.

The obtained submicrometre spherical particles were characterized by a field-emission scanning electron microscope (FE-SEM) (JEOL, JSM-7001FA, Akishima, Japan), transmission electron microscope (TEM) (FEI, Titan3 G2 60-300, Hillsboro, OR, USA) and x-ray diffractometer (XRD) (Rigaku, Ultima IV, Akishima, Japan). The zeta potential of colloidal solutions was measured by a zeta potential/particle size analyser (Beckman Coulter, DelsaNano HC, Brea, CA, USA).

## 3. Results and Discussion

### 3.1. Au Concentration Dependence on Internal Structure

Before laser irradiation, the zeta potential of the mixed solution of Au and Fe_2_O_3_ raw nanoparticles with the different weight ratios was measured. Figure S1 (in Supporting Information) indicates that the absolute values of the zeta potential became closer to zero from those of end members in most of the concentration ranges; hence, particles possibly tend to aggregate or become unstable and the immediacy of two constituent particles was ensured—conditions that are suitable for submicrometre spherical particle fabrication by PLML. 

**Figure 2:**
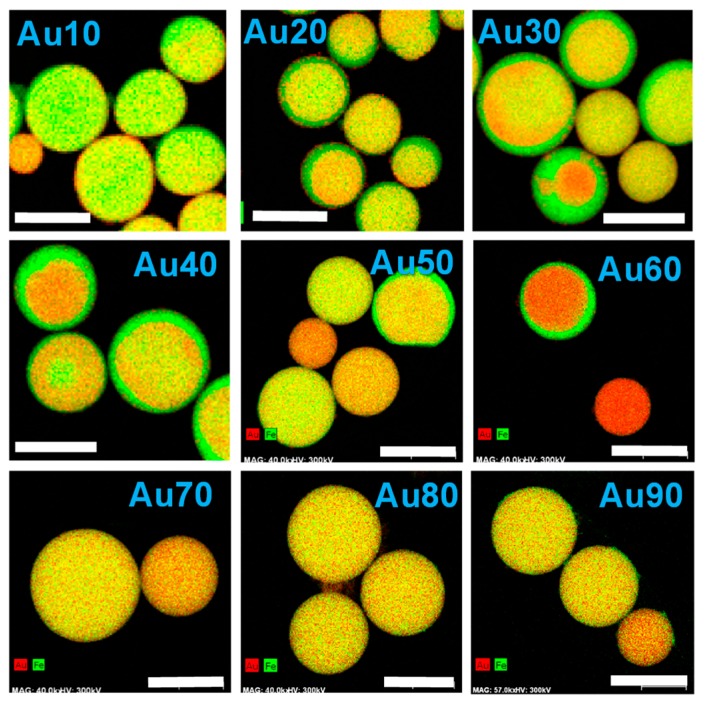
EDS mapping images of particles obtained with different mixing ratios of raw nanoparticle solutions of Au and Fe_2_O_3_. “Au10” indicates an Au 10%: Fe 90% in weight. The red and green colours correspond to the Au and Fe components, respectively. Corresponding HAADF-STEM images are shown in Figure S2 in Supporting Information. All scale bars are 500 nm.

Figure 2 and Figure S2 depict energy-dispersive x-ray spectroscopy (EDS) mapping of Au (red) and Fe (green) and corresponding TEM images of the particles obtained from the raw nanoparticle solution with different mixing ratios. The images in Figure S2 are taken in the high-angle annular dark-field scanning transmission electron microscopy (HAADF-STEM) mode and therefore heavier atoms are brightly indicated, unlike conventional TEM images. Thus, Fe-rich regions (green) in Figure 2 correspond to the dark parts in Figure S2, while Au-rich regions (red) correspond to the bright parts. These figures clearly indicate that the compositional distribution in the particles in Figure 2 corresponded well to the contrast in HAADF-STEM images in Figure S2. Submicrometre spherical particles were formed in all concentration ranges, from Au10 to Au90. From Au10 to Au60, compositional inhomogeneity, mainly in a core (Au-rich)/shell (Fe-rich) structure, was observed. This structure is totally opposite to the core (Fe-rich)/shell (Au-rich)-structured nanoparticles obtained by PLAL [36], which will be discussed later. However, compositionally homogeneous particles were obtained from Au70 to Au90, indicating Au-Fe alloy formation. On the basis of these images, we also found that larger particles tend to be more phase-separated than smaller particles. 

**Figure 3:**
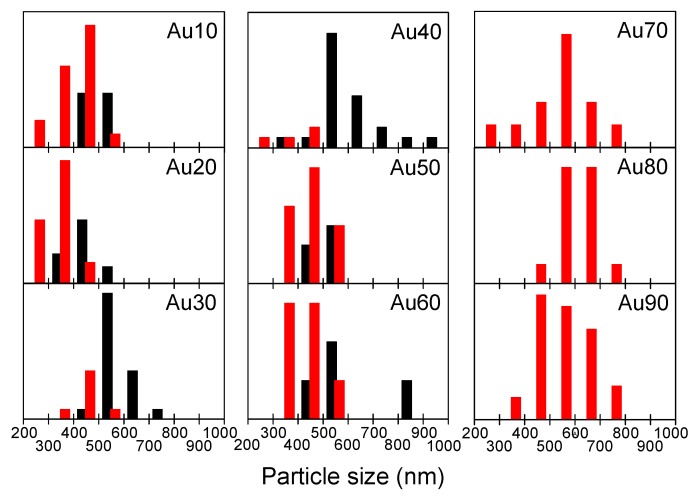
Size histograms of the particles obtained with different mixing ratios of the raw nanoparticle solution of Au and Fe_2_O_3_. “Au10” denotes Au 10%:Fe 90% in weight. The black and red bars indicate the frequencies of phase-separated and homogeneous submicrometre particles in every 100 nm step.

Figure 3 depicts the size histograms of the phase-separated particles (black) and homogeneous particles (red) obtained from Figure 2 and Figure S2. Bars placed in the size range between 400–500 nm indicate that the black bar is the frequency for phase-separated particles 400–500 nm in diameter and the red bar is the frequency of homogeneous particles 400–500 nm in diameter. The phase-separated particles (mainly in the core/shell structure) were observed within the Au10–Au60 range, especially in larger particles, whereas homogeneous particles were observed in the smaller size range. In the Au70–Au90 range, all the particles observed were compositionally homogeneous. 

**Figure 4:**
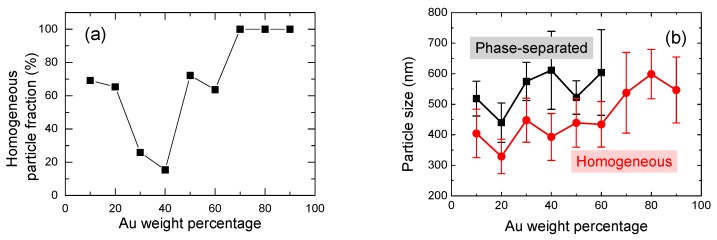
(**a**) Homogeneous particle fraction as a function of Au weight percentage. (**b**) Average particle size of phase-separated and homogeneous submicrometre particles as a function of Au weight percentage.

Figure 4a shows the homogeneous particle fraction as a function of Au weight percentage. Homogeneous particles for Au30 and Au40 were less abundant. Figure 4b shows the change in average particle diameter of homogeneous and phase-separated submicrometre particles. Even though the relative ratio of phase-separated and homogeneous particles drastically changes with Au weight percentage, as shown in Figure 4a, the average size of both particles gradually increased with the Au content and the size difference between them—about 150 nm—was almost constant across all content ranges. 

**Figure 5:**
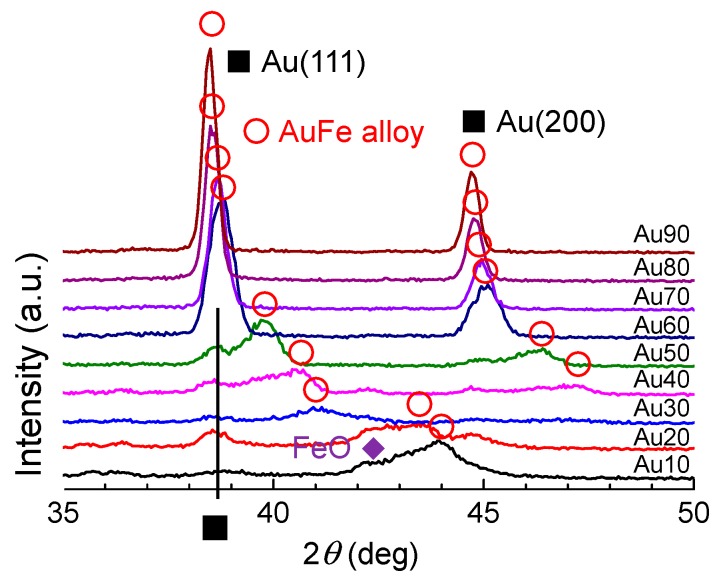
XRD patterns of particles obtained by laser irradiation onto the mixed colloidal solution of Au and Fe_2_O_3_ in ethanol. “Au10” denotes Au 10%:Fe 90% in weight.

Figure 5 presents XRD patterns of the obtained submicrometre spherical particles. Peaks with open circles indicate face-centred cubic (fcc) AuFe alloys with the end members of fcc-Au and fcc-γ-Fe, which were observed in all mixing ratio ranges. The peak positions of AuFe alloys shifted with the change in Au:Fe mixing ratios. Furthermore, Au peaks from raw particles were also detected from Au10–Au50, suggesting that the phase-separated particles are composed of nearly pure Au metal and Fe-rich Au-Fe alloys in this range. FeO peaks were also observed at the Fe-rich side of Au10 and Au20.

**Figure 6:**
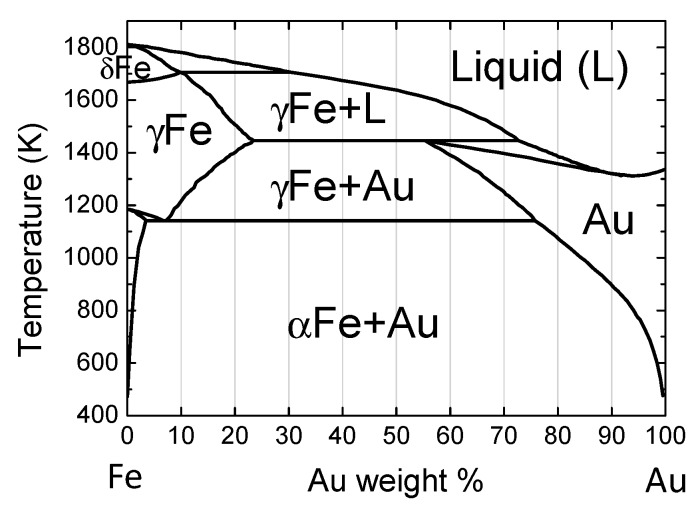
Phase diagram of Au-Fe system.

Figure 6 provides the phase diagram of the Au-Fe system redrawn from [43]. On the basis of the horizontal line at 1446 K between Au20 and Au70, the fcc γ-Fe phase (Fe-rich solid solution) is segregated, while Au is still in its melting phase at the initial stage of solidification. Because of the rapid quenching process, phase-separated submicrometre particles composed of fcc Au-rich alloy phases and fcc γ-Fe-rich alloy phases were thus formed. 

**Figure 7:**
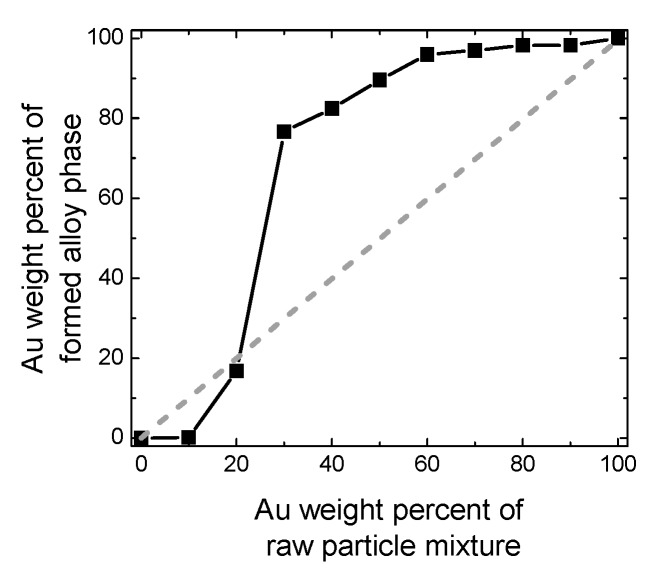
Relationship between the Au weight percentage of the raw particle solution and that of the formed alloy phase after laser irradiation.

From the XRD peak shift of the Au-Fe alloy in Figure 5, the composition change of the alloy phase can be estimated, assuming that Vegard’s law is true for this system. Figure 7 plots the Au weight percent in the alloy phase, estimated as a function of the weight percent of Au raw particles. The estimated Au content values of produced particles showed a content gap between Au 20 to 70 weight percent, which nearly corresponded to the immiscible gap in Figure 6. However, the generated Au-rich alloy phase has a higher Au content than the feeding composition ratio of the raw particle mixture, suggesting that Fe is removed from the product during the PLML process. This is also supported from yellow colour of supernatant probably from ferric ion during the centrifugation process for XRD sample preparation.

### 3.2. Time Dependence of Produced Particles

**Figure 8:**
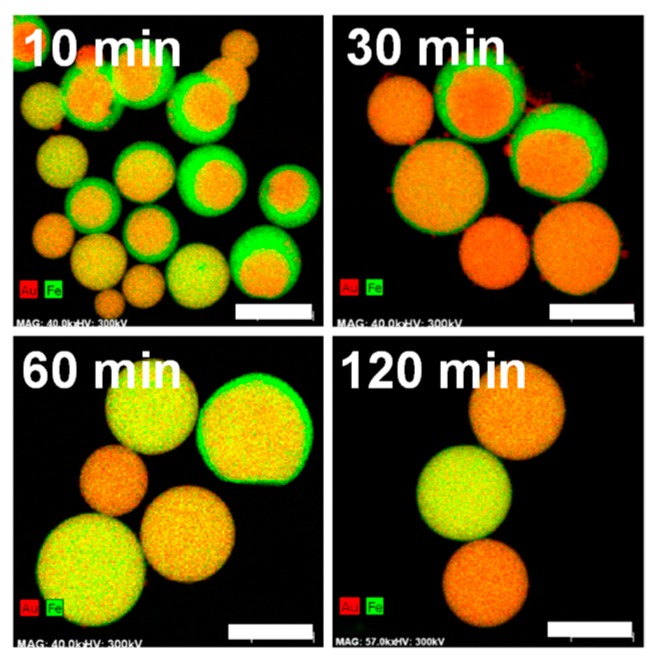
EDS mapping images of particles obtained by different laser irradiation times from the Au50 raw particle solution. Corresponding HAADF-STEM images are shown in Figure S3 in Supporting Information. All scale bars are 500 nm.

Figure 8 and Figure S3 show a plot of the laser irradiation time dependence of elemental mapping and HAADF-STEM images of submicrometre spherical particles obtained by PLML at Au50, respectively. These figures clearly indicate a compositional inhomogeneity in the core (Au-rich)/shell (Fe-rich) structure at short laser irradiation times and a gradual homogenization of the particles by extending the laser irradiation time. The particle size also became larger as irradiation time increased. 

**Figure 9:**
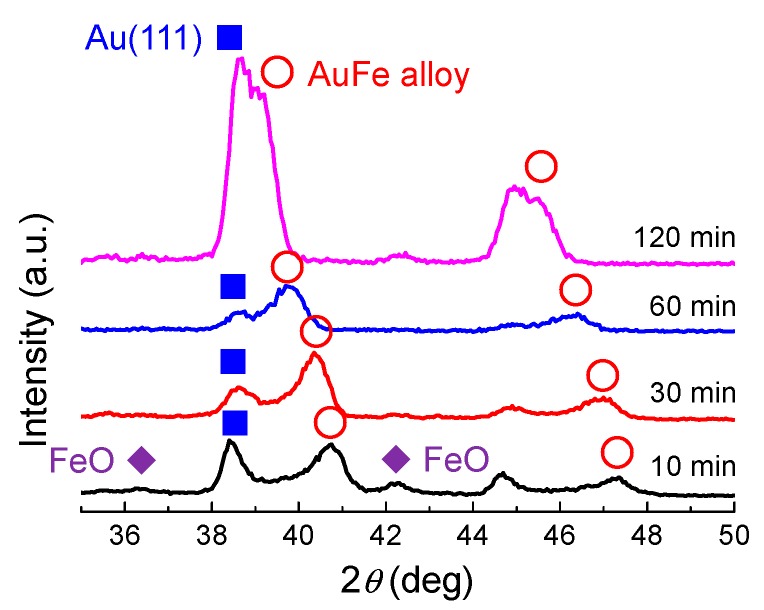
XRD patterns of particles obtained by different laser irradiation time onto the mixed colloidal solution of Au50 in ethanol.

Figure 9 shows the XRD patterns of particles obtained by different laser irradiation times onto mixed colloidal solutions of Au50 in ethanol. FeO and Au peaks originating from the raw particles were observed by 10 min laser irradiation, together with the Au-Fe alloy peak. These FeO and Au peaks gradually became smaller by extending the laser irradiation time, implying that the particles were merging and reacting. Peaks from the AuFe alloy (marked as open circles) shifted to pure Au peaks with the increase in laser irradiation time. These results suggest that the Fe component was gradually removed from the alloy system. 

Figure S4 shows the laser irradiation time dependence of the average Au weight percentage of particles measured by EDS. When the irradiation time was 10 min, the Au content was 57.4% in weight, close to the value of the raw particle solution. However, by extending the irradiation time, the percentage of Au increased while that of Fe decreased, indicating a dissolution of the Fe component. This indication is also supported by the colour change of the solution to light yellow, the typical colour of the Fe ion. 

### 3.3. Comparison with Au-Fe Nanoparticles and Formation Mechanism

In the case of Au-Fe nanoparticles obtained by PLAL, Fe-core/Au-shell nanoparticles are usually obtained, in which the core and shell combination is contrary to our case. In the nanoparticle case, the surface area becomes large and the surface energy can dominate the stabilization process of the Fe-core/Au-shell structure because of the low surface energy of Au and short diffusion distance of the nanoparticles [36]. In contrast, in submicrometre particles obtained by PLML, the bulk thermodynamic contribution is dominant, resulting in the solidification of the Fe-rich component at the surface and Au enrichment in the core, as deduced from the phase diagram in Figure 6.

**Figure 10:**
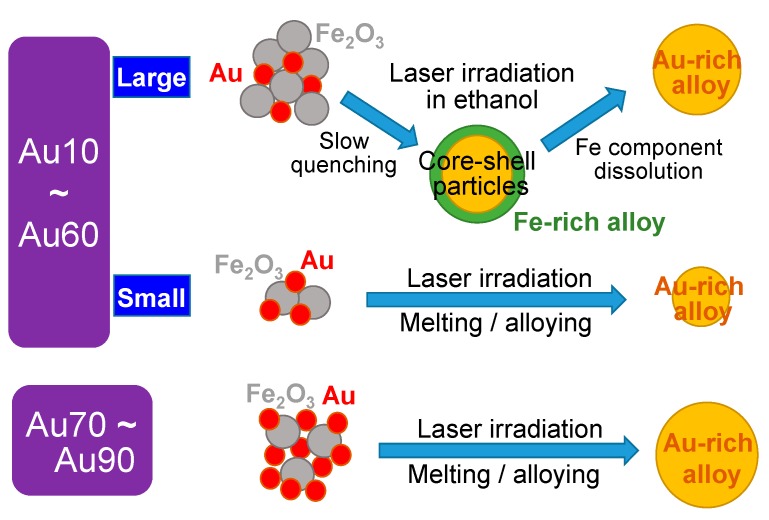
Schematic illustration of the Au-Fe submicrometre spherical particle formation process by PLML in ethanol.

Figure 10 summarizes the formation mechanism of submicrometre spherical particles by PLML. Particles marked in red and in grey are well-aggregated Au and Fe_2_O_3_ raw nanoparticles. The yellow and green areas correspond to Au-rich and Fe-rich Au-Fe alloys.

In the case of Au10–Au60 with large particle sizes, the raw particles melt, merge with each other and quench to form alloy particles that are submicrometre in size. However, in this content range on the phase diagram, particles should be phase-separated, with the segregation of Fe-rich AuFe alloy phases at the particle surface because quenching induces an earlier solidification of the Fe-rich component by cooling from the surrounding ethanol. The Au component is pushed toward the centre direction during quenching. The core-shell structure, containing an Au-rich core and Fe-rich shell, is generated by this process. Further laser irradiation induces the thermochemical dissolution of the Fe component to ethanol and the enrichment of Au content in the remaining Au-rich alloy particles. 

In the case of Au10–Au60 with small particle sizes, the particles should be phase-separated from the phase diagram, as above. However, the quenching rate is quite fast, owing to the small particle size and particles are solidified before phase separation occurs. Thus, homogeneous particles that might be in an amorphous phase were fabricated. In the case of Au70–Au90, Au-rich homogeneous particles without phase separation were generated, following the phase diagram. When the particle size was small, the particles tended to be single-crystalline by the rapid shifting of the crystallization front. 

Previously, our group reported Au-Fe submicrometre spherical particle formation using the 532 nm Nd:YAG laser and chemically fabricated Fe_3_O_4_ nanoparticles under slightly different laser irradiation conditions (100 mJ, 30 Hz, 60 min) [22]. Raw particles with different relative atomic ratios of Au:Fe—10:1, 1:1, 1:10 (corresponding to Au97, Au78 and Au26 in our notation of the Au-Fe alloy system based on weight percent)—were used as starting materials and the products after laser irradiation were characterized by XRD. At Au97, a nearly Au metal peak was observed, as expected. In contrast, Au metal, broad Au-Fe alloy and FeO peaks were observed at Au78; Au metal, raw Fe_3_O_4_ and partially reduced FeO peaks were observed at Au26. In both cases, raw and slightly reduced Fe components (FeO) were observed, indicating the insufficient reduction reaction, because these components could not be observed in this experiment. This insufficiency in the previous work might be the effect of a lower fluence, relatively inhomogeneous heating and the less intimacy of raw particles during laser irradiation. 

Thus, by adopting a 355 nm laser to enhance homogeneous heating and by selecting raw particles with appropriate surface charge to promote interparticle immediacy, we can predict most of the products by the phase diagram obtained under thermodynamic equilibrium. The only exception in this case was the formation of homogeneous particles smaller than 450 nm for Au 10–Au60 that resulted from the rapid quenching rate of 10^10^ K s^−1^. 

By repetitive laser irradiation, the Au:Fe composition ratio gradually approached the thermodynamically possible alloy composition based on the time-dependence of the products. In contrast, in order to obtain non-equilibrium alloy particles, opposite measures adopted in this study, such as inhomogeneous heating and less encounters between constituent particles, will probably be effective. 

## 4. Conclusions

We studied the reactive fabrication of submicrometre spherical particles combining Au and Fe by PLML using a 355 nm Nd:YAG laser to induce more homogeneous heating of constituent particles and Fe_2_O_3_ nanoparticles as a raw iron source to promote contact between Au and Fe in liquid. The particles obtained by this process can be explained almost on the basis of the phase diagram whether they are homogeneous or phase-separated. For the content range where Au-Fe phase has to be separated, Fe component is enriched at the surface due to the quenching from the surface for larger particles, while smaller particles tend to be homogeneous particles due to the rapid quenching without crystallization. If we extend laser irradiation time, particles approach the thermodynamically stable compositions. Therefore, in order to fabricate submicrometre alloy particles of immiscible combination, counter measures to this study, inhomogeneous heating and less contact are preferable. 

## Supplementary Materials

The following are available online at http://www.mdpi.com/2079-4991/9/2/198/s1, Figure S1: Zeta potential of the mixed solution of Au and Fe_2_O_3_ nanoparticles before laser irradiation, Figure S2: HAADF-STEM images of particles obtained with different mixing ratios of raw nanoparticle solutions of Au and Fe_2_O_3_, Figure S3: HAADF-STEM images of particles obtained by different laser irradiation times from the Au50 raw particle solution, Figure S4: Au weight percent change in the produced submicrometre particles with the laser irradiation time for Au50.
