# Supplementary material for: Determining the Composite Structure of Au-Fe-Based Submicrometre Spherical Particles Fabricated by Pulsed-Laser Melting in Liquid
Source: Nanomaterials (Basel). 2019 Feb 3;9(2):198. doi: 10.3390/nano9020198 (PMC6409745; doi:10.3390/nano9020198)
Supplement: Supplementary file 1 [file nanomaterials-09-00198-s001.pdf]

Supporting Information

Determining the composite structure of Au-Fe-based submicrometer spherical particles fabricated by pulsed-laser melting in liquid

Hokuto Fuse [1], Naoto Koshizaki [1*], Yoshie Ishikawa [2], and Zaneta Swiatkowska-Warkocka[3]

[1] Graduate School of Engineering, Hokkaido University, Sapporo, Hokkaido 060-8628, Japan; ttx-ken@frontier.hokudai.ac.jp, koshizaki.naoto@eng.hokudai.ac.jp

[2] Nanomaterials Research Institute, National Institute of Advanced Industrial Science and Technology (AIST), Tsukuba, Ibaraki 305-8565, Japan; ishikawa.yoshie@aist.go.jp

[3] Institute of Nuclear Physics, Polish Academy of Sciences, Kraków, PL-31342, Poland; swiatkow@wp.pl

* Correspondence: koshizaki.naoto@eng.hokudai.ac.jp, +81-11-706-5594

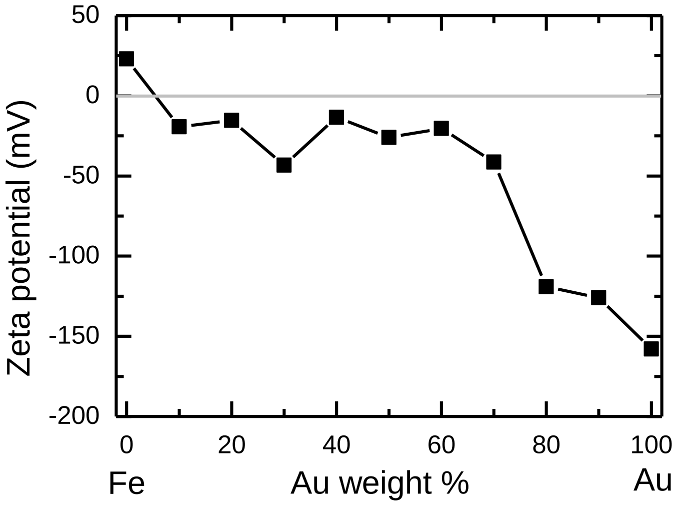

Figure S1. Zeta potential of the mixed solution of Au and Fe_2O_3 nanoparticles before laser irradiation. Most of the concentration range zeta potentials were from −50 mV to 0 mV.

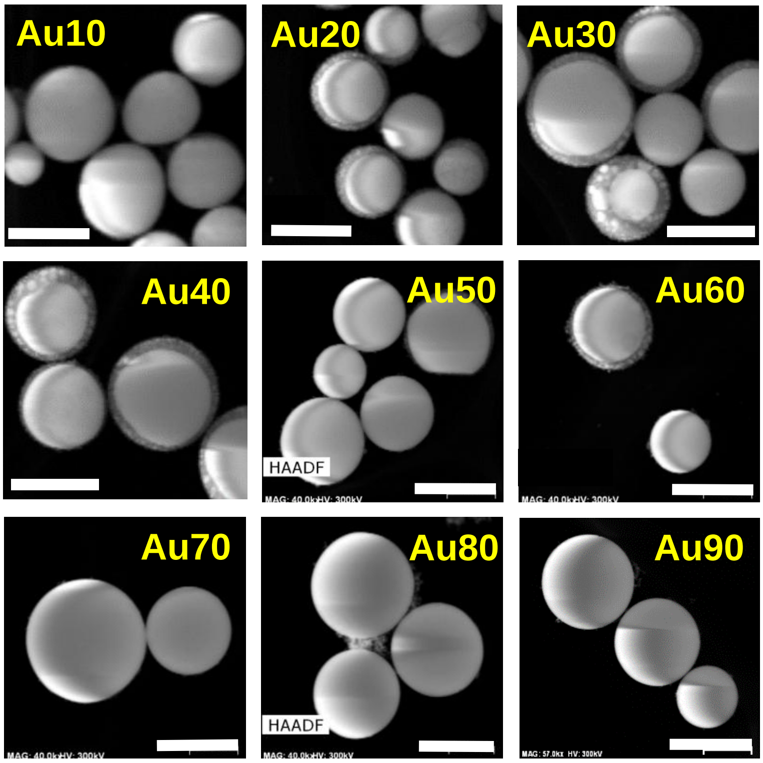

Figure S2. HAADF-STEM images of particles obtained with different mixing ratios of raw nanoparticle solutions of Au and Fe_2O_3. “Au10” indicates Au 10%:Fe 90% in weight. All scale bars are 500 nm.

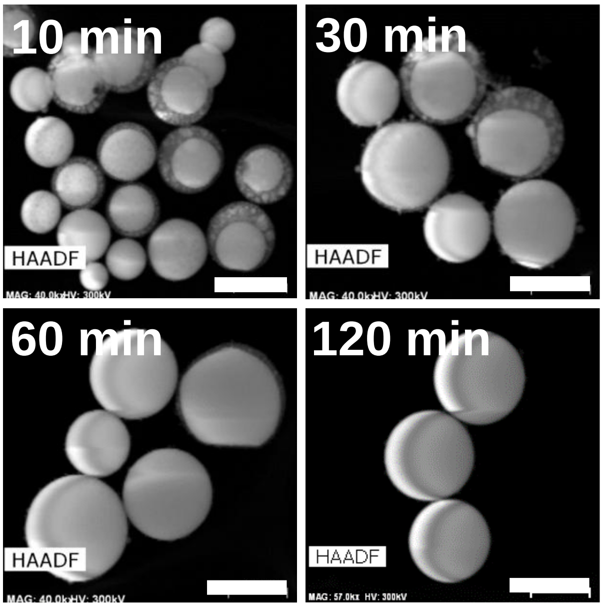

Figure S3. HAADF-STEM images of particles obtained by different laser irradiation times from the Au50 raw particle solution. All scale bars are 500 nm.

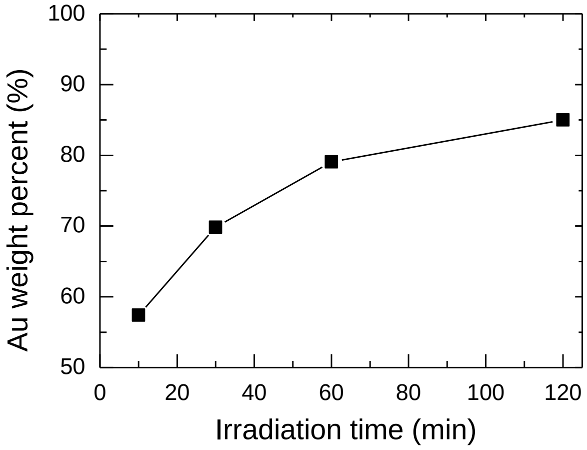

Figure S4. Au weight percent change in the produced submicrometer particles with the laser irradiation time for Au50.
